# Roles of the TGF-β–VEGF-C Pathway in Fibrosis-Related Lymphangiogenesis

**DOI:** 10.3390/ijms19092487

**Published:** 2018-08-23

**Authors:** Hiroshi Kinashi, Yasuhiko Ito, Ting Sun, Takayuki Katsuno, Yoshifumi Takei

**Affiliations:** 1 Department of Nephrology and Rheumatology, Aichi Medical University, Nagakute 480-1195, Japan; hiroshi.kinashi@gmail.com (H.K.); t-katsuno@aichi-med-u.ac.jp (T.K.); 2 Department of Nephrology and Renal Replacement Therapy, Nagoya University Graduate School of Medicine, Nagoya 466-8550, Japan; tinasuen1991@gmail.com; 3 Department of Medicinal Biochemistry, School of Pharmacy, Aichi Gakuin University, Nagoya 464-8650, Japan; takei@dpc.agu.ac.jp

**Keywords:** lymphangiogenesis, fibrosis, transforming growth factor-β, vascular endothelial growth factor-C

## Abstract

Lymphatic vessels drain excess tissue fluids to maintain the interstitial environment. Lymphatic capillaries develop during the progression of tissue fibrosis in various clinical and pathological situations, such as chronic kidney disease, peritoneal injury during peritoneal dialysis, tissue inflammation, and tumor progression. The role of fibrosis-related lymphangiogenesis appears to vary based on organ specificity and etiology. Signaling via vascular endothelial growth factor (VEGF)-C, VEGF-D, and VEGF receptor (VEGFR)-3 is a central molecular mechanism for lymphangiogenesis. Transforming growth factor-β (TGF-β) is a key player in tissue fibrosis. TGF-β induces peritoneal fibrosis in association with peritoneal dialysis, and also induces peritoneal neoangiogenesis through interaction with VEGF-A. On the other hand, TGF-β has a direct inhibitory effect on lymphatic endothelial cell growth. We proposed a possible mechanism of the TGF-β–VEGF-C pathway in which TGF-β promotes VEGF-C production in tubular epithelial cells, macrophages, and mesothelial cells, leading to lymphangiogenesis in renal and peritoneal fibrosis. Connective tissue growth factor (CTGF) is also involved in fibrosis-associated renal lymphangiogenesis through interaction with VEGF-C, in part by mediating TGF-β signaling. Further clarification of the mechanism might lead to the development of new therapeutic strategies to treat fibrotic diseases.

## 1. Introduction

**Figure 1:**
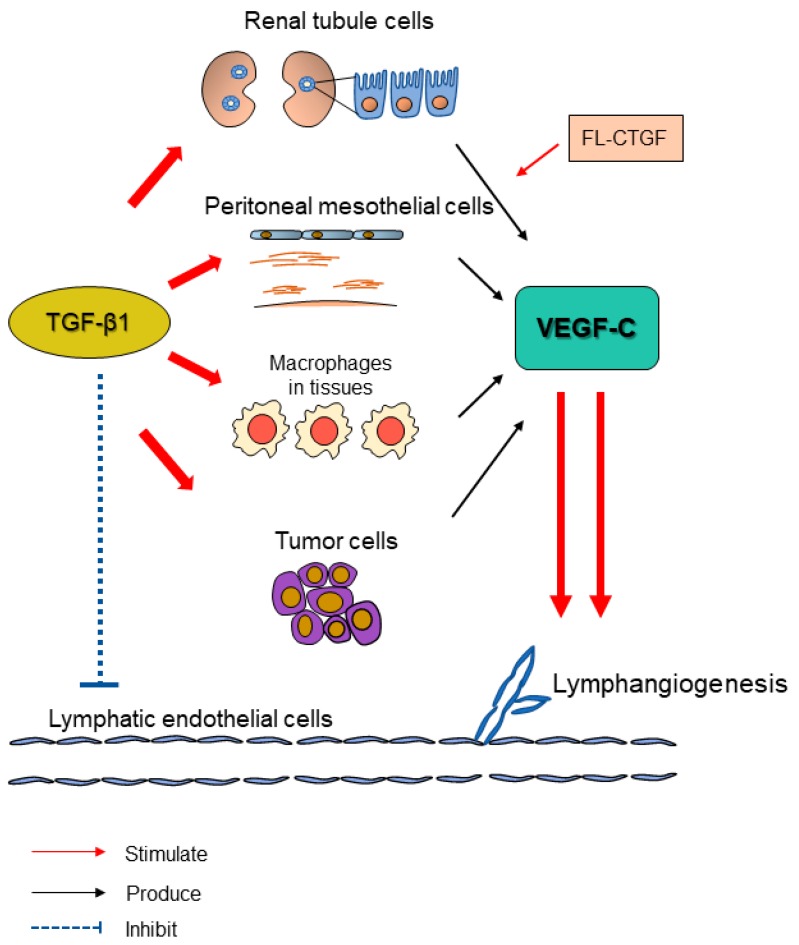
Transforming growth factor-β (TGF-β) promotes tissue lymphangiogenesis via upregulating vascular endothelial growth factor (VEGF)-C in various fibrotic pathologies (kidney fibrosis, peritoneal fibrosis, tissue inflammation, and tumor microenvironment). Lymphangiogenesis is generally observed during tissue fibrosis. One possible mechanism is acknowledged to be the TGF-β–VEGF-C pathway. TGF-β, as a key player in tissue fibrosis, is demonstrated to have a direct inhibitory effect on the proliferation and migration of lymphatic endothelial cells (LECs) [12]. However, TGF-β is found to enhance VEGF-C production in renal proximal tubule cells [8,9], collecting tubule cells [8], peritoneal mesothelial cells [10], macrophages [8,9,10], and some tumor cells [15]. The upregulated VEGF-C increases the growth and tube formation of LECs, abrogating the inhibitory effects of TGF-β and leading to lymphangiogenesis. Additionally, connective tissue growth factor (CTGF), as induced by TGF-β in multiple kinds of cells, also promotes the production of VEGF-C during renal fibrosis [16]. Full-length CTGF binds to VEGF-C and suppresses the tube formation of LECs while the effect is counteracted by the cleavage of CTGF in vivo [16]. In summary, increased TGF-β levels promote VEGF-C production in specific cells and lead to lymphangiogenesis during tissue fibrosis.

Lymphatic vessels reabsorb extravasated tissue fluid containing cells and proteins, and return them to the blood circulation, thereby maintaining interstitial homeostasis [1]. The increase of lymphatic capillaries, which is mainly induced by sprouting from basal lymphatics, is known as lymphangiogenesis, and is observed in a variety of diseases such as tumor metastasis [2], inflammation [3], wound healing [4], and organ transplantation [5]. Signals mediated by vascular endothelial growth factor (VEGF)-C, VEGF-D, and its receptor VEGF receptor (VEGFR)-3 are recognized as a central molecular mechanism of lymphangiogenesis [6,7]. Lymphangiogenesis also occurs during the development of fibrosis in several organs, including kidney and peritoneum [8,9,10]. Among various molecular mechanisms, transforming growth factor-β (TGF-β) plays a central role in tissue fibrosis [11]. Regarding the role of TGF-β in lymphangiogenesis, several studies showed that TGF-β has an inhibitory effect on the growth of lymphatic endothelial cells (LECs) [12,13,14]. However, we suggested the possible involvement of the TGF-β–VEGF-C pathway, by which TGF-β promotes VEGF-C production in several cell types, leading to lymphangiogenesis in renal and peritoneal fibrosis [8,10]. This review is focusing on some pathologies including renal fibrosis, peritoneal fibrosis (during peritoneal dialysis), tissue inflammation, and tumor progression, as summarized in Figure 1.

## 2. Induction of Peritoneal Fibrosis and Neoangiogenesis by TGF-β

Long-term peritoneal dialysis (PD) treatment results in submesothelial fibrosis and neoangiogenesis, which is accompanied by high peritoneal solute transport and the loss of ultrafiltration. PD treatment induces the production of pro-inflammatory cytokines and growth factors, and epithelial–mesenchymal transition (EMT) in peritoneal mesothelial cells. Several factors such as uremia, exposure to dialysate, and peritonitis episodes cause peritoneal fibrosis, which is mainly mediated by TGF-β [10]. Neoangiogenesis and high vascular permeability are induced by VEGF-A and pro-inflammatory cytokines, which contribute to high peritoneal solute transport and ultrafiltration failure (UFF).

Mesothelial cells that cover the surface of the peritoneal membrane provide local defense and predominantly regulate peritoneal homeostasis, including the synthesis of cytokines, growth factors, and matrix proteins [17,18]. Prolonged PD induces mesothelial cell activation, cell hypertrophy, and some degree of mesothelial denudation [19]. Glucose, an osmotic agent of the dialysate, inhibits mesothelial cell proliferation [20], and induces mitochondrial DNA damage and the apoptosis of mesothelial cells [21,22]. Glucose degradation products (GDPs) are also toxic for cells in the peritoneum [23]. Glucose and GDPs increase the production of inflammatory cytokines and growth factors in mesothelial cells, such as monocyte chemoattractant protein-1 (MCP-1) [24,25,26], TGF-β [26,27], and VEGF-A [28]. Furthermore, PD treatment induces the loss of epithelial phenotype and the further acquisition of mesenchymal characteristics by mesothelial cells, which is initiated by profibrotic and inflammatory cytokines, including TGF-β1 and interleukin (IL)-1β [29].

Peritonitis is an important cause of peritoneal membrane injury, which leads to peritoneal fibrosis, neoangiogenesis, and peritoneal dysfunction [19]. Peritonitis causes the exfoliation of mesothelial cells, disruption of the underlying basement membrane, and the loss of microvilli and cell-cell contact in the remaining mesothelial cells [30]. Severe peritonitis induces the exudation of fibrin on the surface of the peritoneum, and is accompanied by inflammatory cell infiltration [31]. Bacterial peritonitis rapidly increases the total number of cells in the peritoneum, including neutrophils and macrophages, which can last for several weeks after the clinical remission of peritonitis. Similarly, pro-inflammatory cytokines and fibrogenic growth factors such as IL-1β, IL-6, TGF-β, and fibroblast growth factors (FGF) were increased through at least six weeks despite the clinical resolution of peritonitis [32].

Myofibroblasts, which play an important role in peritoneal fibrosis, can derive from different origins such as resident fibroblasts, endothelial cells, and bone marrow-derived cells [33]. Glucose in the dialysis solution stimulates fibroblast proliferation with the increased secretion of extracellular matrix (ECM) proteins, leading to peritoneal fibrosis [34,35,36]. Mesothelial cells can also directly contribute to fibrosis by undergoing EMT followed by migration into the submesothelium and the production of ECM [29]. PD patients with peritoneal fibrosis showed high levels of TGF-β/Smad signaling, which activates the transcription of target genes and induces EMT [37,38]. The blocking of TGF-β signaling by specific peptides reduced fibrosis, neoangiogenesis, and EMT, and improved peritoneal function in a rodent model [33].

Peritoneal capillaries are embedded in a loose adipose layer under the submesothelium [39]. Peritoneal neoangiogenesis and high vascular permeability cause an increase of small solute transport, and is accompanied by a rapid reduction of glucose-driven osmotic pressure, which contributes to a loss of ultrafiltration. High blood vessel density and vascular subendothelial hyalinization were observed in the peritoneum of patients with membrane failure [19]. The exposure to glucose-based dialysate results in the deposition of advanced glycation end products (AGE) in the peritoneal interstitium and microvascular walls of PD patients. AGE accumulation was correlated with interstitial fibrosis, vascular sclerosis, and impaired ultrafiltration [40].

VEGF-A is a central regulator of angiogenesis and vascular permeability [41]. VEGF-A expression in human PD effluents was correlated with peritoneal permeability for small solutes and the loss of ultrafiltration [42]. GDPs induce VEGF-A production in mesothelial cells and endothelial cells [28]. AGE induces angiogenesis through the induction of autocrine signaling VEGF-A in microvascular endothelial cells [43]. Mesothelial cells that have undergone EMT produced higher amounts of VEGF-A than normal epithelial-differentiated mesothelial cells [44]. 

The development of peritoneal neoangiogenesis and fibrosis is mediated by the close interaction of TGF-β and VEGF-A. The overexpression of TGF-β1 induced rat peritoneal fibrosis, accompanied by neoangiogenesis, through the induction of VEGF-A production in mesothelial cells [45]. We recently evaluated the regulatory mechanisms between TGF-β1 and VEGF-A in PD [46]. There was a positive correlation between TGF-β1 and VEGF-A expression in human PD effluents. In vitro, TGF-β1-induced VEGF-A upregulation in cultured mesothelial cells and fibroblasts, which was specifically suppressed by treatment with a TGF-β type I receptor inhibitor. In vivo, VEGF-A induction and neoangiogenesis in chlorhexidine gluconate (CHG)-induced rat peritoneal fibrosis models were clearly suppressed by a TGF-β type I receptor inhibitor. These results suggest the possible involvement of the TGF-β1–VEGF-A pathway in fibrosis-associated peritoneal neoangiogenesis.

## 3. Roles of Renal and Peritoneal Lymphatics

Lymphatic vessels drain extravasated tissue fluid and return it to the blood circulation, thereby maintaining interstitial fluid homeostasis [1]. In addition, dietary lipids in the intestine are absorbed by lymphatic vessels [7]. The obstruction of main lymphatic ducts results in the backflow of lymph and the appearance of chyluria, revealing the connection between systemic lymphatics, renal lymphatics, and the urinary tract [47]. Lymphatics also function as a trafficking route for immune cells [7]. In normal kidney tissue, lymphatic vessels are localized around blood vessels, and are greater in size than interlobular arteries [48,49]. In human kidney transplant rejection, a prominent increase of lymphatic vessels was observed within nodular mononuclear infiltrates, and it is speculated that lymphatic vessels not only drain inflammatory infiltrate, but also maintain immune responses by producing lymphatic chemokines that attracts inflammatory cells [48]. Donor-derived macrophages might differentiate into LECs, which contribute to increase the number of lymphatic vessels in human renal transplants [50]. Aminopeptidase p and podoplanin are discriminatory markers for vascular and lymphatic endothelial cells in rat remnant kidneys, and the proliferation of lymphatic vessels was observed in tubulointerstitial fibrotic areas, accompanied by the rarefaction of blood vessels [51]. Human kidney biopsies, such as for diabetic nephropathy, showed an increase of lymphatic vessels in tubulointerstitial injured areas. The expression of lymphatic vessels was correlated with the degree of inflammation and fibrosis of kidney tissue; a stronger correlation was observed for fibrosis [49].

It might be difficult to show the general therapeutic strategy for the regulation of lymphangiogenesis in kidney diseases. Mammalian target of rapamycin (mTOR) inhibitors are known to impede LEC growth through the impairment of VEGF-C downstream signaling [52]. In renal transplantation models, the mTOR inhibitor sirolimus inhibited lymphangiogenesis in association with an attenuated development of chronic kidney allograft injury [53]. However, the specific blocking of lymphangiogenesis by an anti-VEGFR-3 antibody did not prevent inflammation, interstitial fibrosis, and proteinuria in a rat adriamycin-induced proteinuric nephropathy model [54]. In contrast, the further induction of lymphangiogenesis by VEGF-C treatment suppressed inflammatory infiltrates and reduced inflammatory cytokines and TGF-β1 expression, leading to attenuate renal fibrosis in the mouse model of unilateral ureteral obstruction [55]. Interestingly, accelerated lymphangiogenesis by the administration of VEGF-C protein reduced the infiltration of M2 macrophages, which inhibited cyst formation and improved decreased renal function in a mouse polycystic kidney model [56]. Thus, the requirement for lymphangiogenesis and the efficacy of therapeutic intervention vary depending on disease etiology, and further studies are needed to clarify the role of renal lymphatics in a variety of kidney diseases.

Peritoneal lymphatic vessels continuously absorb dialysate during PD treatment, which reduces effective ultrafiltration [57]. It is known that the diaphragm contains a specialized lymphatic absorption system that includes lymphatic lacunae and mesothelial stomata, and plays a central role in peritoneal lymphatic absorption [58]. More than a decade ago, clinical studies showed that increased lymphatic absorption was related to long-term PD and UFF [59,60]. However, since then, the accuracy of estimated lymphatic absorption, which is based on the disappearance rate of intraperitoneally administered macromolecules such as radioactive iodinated serum albumin or dextran, has generated considerable debate [61,62]. Subsequently, there has been a reduced focus on the lymphatics of PD.

However, we suggested the possible involvement of peritoneal lymphangiogenesis in the mechanism of UFF in PD-related peritoneal fibrosis. The VEGF-C protein level in human PD effluents was increased in association with high peritoneal solute transport, and was correlated with the TGF-β1 concentrations in PD effluents [10,63]. VEGF-C mRNA expression and lymphatic markers were increased in the peritoneal biopsies of UFF patients, and were correlated with peritoneal thickness [10]. VEGF-C expression was mainly upregulated in mesothelial cells and macrophages in human peritoneal biopsies with bacterial peritonitis. In addition, VEGF-C expression in cultured mesothelial cells and macrophages was increased by TGF-β1 treatment [8,10]. In the rat peritoneal fibrosis model induced by CHG, the expression of lymphatic vessel markers and VEGF-C was increased, and it was accompanied by peritoneal inflammation and fibrosis [10]. Interestingly, dilated giant lymphatic vessels were induced in the diaphragm in association with CHG-induced peritoneal fibrosis. The increased expression of lymphatic vessel markers and VEGF-C was reduced by treatment with a TGF-β type I receptor inhibitor [10]. Treatment with a cyclooxygenase-2 (COX-2) inhibitor also reduced peritoneal lymphangiogenesis in an experimental PD model [10,64], which might be mediated by reduced VEGF-C production in macrophages [65]. Interestingly, in the rat remnant kidney model, it was demonstrated that chronic kidney disease itself induced peritoneal fibrosis, lymphangiogenesis, and a high lymphatic absorption rate, independent of exposure to PD solution [66].

Icodextrin is a glucose polymer derived from starch, and is one of the alternatives to glucose as an osmotic agent in peritoneal dialysate. Icodextrin is slowly absorbed from the peritoneal cavity, mainly by lymphatic vessels, because its molecular weight is too large for transport into blood capillaries [67]. Icodextrin solution has been widely used because it reduces the metabolic effects of peritoneal glucose exposure and acts as a stable osmotic gradient for long dwells [67]. Icodextrin solution can provide stable ultrafiltration for long dwells, particularly in patients with high peritoneal solute transport and UFF. However, it has been reported that the icodextrin solution did not improve ultrafiltration in some patients with UFF, possibly as a result of increased lymphatic absorption with lymphangiogenesis [68].

Soluble VEGFR-3, a decoy receptor that traps VEGF-C/D, specifically suppressed lymphangiogenesis without changes in inflammation, fibrosis, and neoangiogenesis in the diaphragm of a mouse peritoneal fibrosis model induced by methylglyoxal, which is a toxic GDP [69]. The inhibition of lymphangiogenesis by soluble VEGFR-3 treatment improved the impaired ultrafiltration of icodextrin solution in the model. In addition, treatment with soluble VEGFR-3 did not change peritoneal solute transport, and tended to increase the impaired ultrafiltration volume of a glucose-based solution [69]. We propose that the development of new PD solutions such as icodextrin might lead to a further focus on the function of peritoneal lymphatic vessels in association with PD treatment.

## 4. Lymphangiogenesis Occurs during Tissue Fibrosis

Composed of lymphatic capillaries, pre-collector lymphatic vessels, and thoracic duct, the lymphatic vasculature functions as a transporter for excessive fluid to return to the blood circulation. The specific expression of lymphatic markers distinguishes lymphatics from blood vessels, including the Prospero-related homeobox transcription factor 1 (Prox1), VEGFR-3, and lymphatic vessel hyaluronan receptor-1 (LYVE-1), which are all highly expressed in lymphatic capillaries. In collector lymphatic vessels, the expression of markers is much lower than capillaries, but levels of Prox1 and VEGFR-3 remain high in the valves [1]. 

The formation of new lymphatic capillaries, which is defined as lymphangiogenesis, has been observed during acute and chronic inflammation, tumor metastasis, and tissue remodeling. On one hand, the unique structure of the vascular walls, which lack pericytes, and the basement membrane of lymphatic capillaries facilitate the absorption of water and macromolecules from the tissue interstitium. Insufficient lymph drainage, which typically occurs after surgeries such as lymph node removal, could cause local fluid retention, known as lymphedema. On the other hand, lymphatic vessels also transport immune cells, including antigen-presenting cells and inflammatory cells such as macrophages and leukocytes, and thus play an active role in innate and adaptive immunity. The specific production of the C–C motif chemokine ligand 21 (CCL21) by LECs bind to C-C chemokine receptor type 7 (CCR7) on dendritic cells (DC), indicating that lymphatics are involved in DC recruitment and migration [70]. Therefore, lymphangiogenesis accompanying tissue injury is crucial for maintaining tissue homeostasis. 

**Figure 2:**
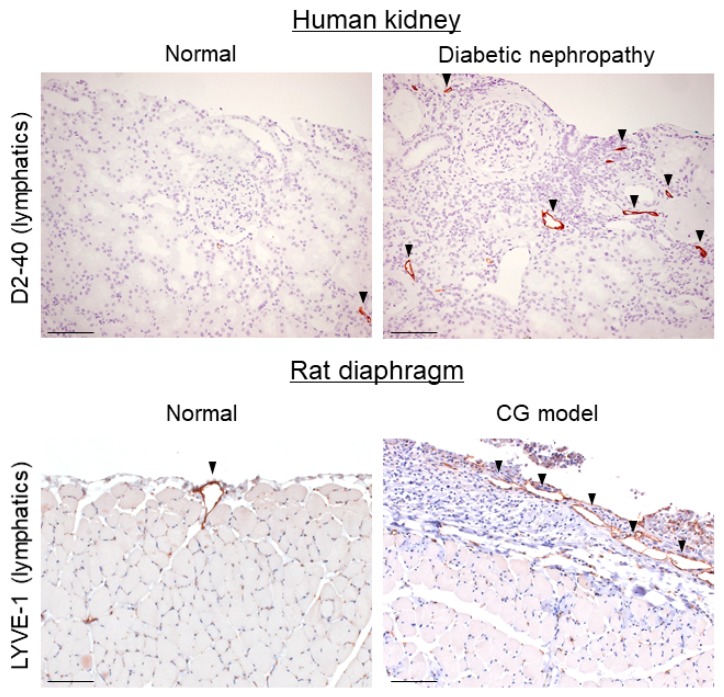
Lymphangiogenesis occurs in renal and peritoneal fibrosis. Lymphangiogenesis was observed in a human diabetic nephropathy case and in a rat diaphragmatic fibrosis model induced by chlorhexidine gluconate (CG) by immunohistochemistry. D2-40 and lymphatic vessel endothelial hyaluronan receptor-1 (LYVE-1) are lymphatic markers. Arrowheads indicate lymphatic vessels. Scale bars = 100 μm.

Lymphangiogenesis is often observed during inflammatory and fibrotic processes in human and animal studies. Increased lymphatic vascular density was observed during myocardial infarction [71,72,73], idiopathic pulmonary fibrosis [74], wound healing [4,75,76], renal fibrosis [8,49,55], peritoneal fibrosis [10,69], tumor fibrosis [77,78] and the like (Table 1) (Figure 2). Fibroblast activation and ECM accumulation lead to tissue repair, remodeling, and a loss of function. The specific structure of lymphatic capillaries allows LECs to associate directly with ECM. Hyaluronic acid, a constituent of ECM, was shown to bind to lymphatic capillaries via its receptor LYVE-1. Moreover, it promoted the proliferation and tube formation of LECs, as well as in vitro lymphangiogenesis [74,79,80]. Besides, molecules regulating the connections between cells and ECM are also related to abnormal lymphatic growth and function, including integrins [81,82], fibronectin extra domain [82], connexins [83], and others.

There are diverse reports regarding the effect of lymphangiogenesis during fibrosis in different tissues and disease models. During peritoneal dialysis, excessive lymphangiogenesis was acknowledged to impair ultrafiltration. The inhibition of lymphangiogenesis by adenovirus-soluble VEGFR-3 improved peritoneal function in a peritoneal fibrosis model [69]. In idiopathic pulmonary fibrosis, lymphangiogenesis was thought to accelerate the disease pathophysiology because of its early appearance and location in normal areas [74]. High CCL21 expression, which came from the hyperplasia of lymphatics, might promote the fibrotic process. However, in a renal fibrosis model induced by unilateral ureteral obstruction (UUO), lymphangiogenesis was thought to play a role in mitigating the fibrosis process [8,9,55]. As well, during skin wound healing, lymphangiogenesis first appeared in the periphery of injured skin, the disturbance of which (by dexamethasone) resulted in delayed wound closure [75]. Additionally, in a mouse tail excision model, fibrosis might hinder the generation of lymphatics, and was a risk factor for lymphedema. Using a collagen gel to reduce fibrosis accelerated lymphatic vessel growth and improved lymphatic function [84].

Although several growth factors are known to induce the initiation of lymphangiogenesis, it is widely acknowledged that the VEGF-C/VEGFR-3 axis plays a determinant role in the process, and others may exert a pro-lymphangiogenic effect via this axis [85,86]. As a tyrosine kinase expressed by the lymphatic endothelium, VEGFR-3 is one of the most used markers of lymphatic vessels, and is indispensable during the development of the cardiovascular system [87,88]. Both VEGF-C and VEGF-D can bind and stimulate VEGFR-3, despite having different structures and undergoing different proteolytic processes [89,90]. Although the overexpression of both factors can promote lymphangiogenesis [91], VEGF-C plays a more vital role in the development of the lymphatic system than does VEGF-D [92,93]. Recently, secreted protein collagen-binding and calcium-binding EGF domains 1 (CCBE-1) has been found to be indispensable during the development of lymphatics. Interestingly, CCBE-1 was involved in the proteolysis of VEGF-C, and was required for VEGF-C function, but not VEGF-D [90,94], suggesting that CCBE-1 could be a novel target in the VEGF-C pathway. 

Changes in VEGF-C expression have been found alongside changes in lymphatics in several studies employing fibrotic models. VEGF-C expression in renal biopsy specimens from patients with kidney disease, especially diabetic nephropathy, was demonstrated to be higher than that of control kidneys. Additionally, VEGF-C was found to be expressed in proximal and collecting tubules [49]. Several reports also illustrated the renal overexpression of VEGF-C after UUO [8,9,55]. Besides tubule cells, infiltrating macrophages were considered to be another main source of VEGF-C in this process [8,9]. Macrophages were also responsible for VEGF-C production in injured tissues including heart [72], skin, tail [4,95], trachea, lung [74,96], peritoneum [10], colon [97], and others. In PD patients, VEGF-C expression was found to be related to peritoneal thickness, and a higher concentration in the dialysate was observed in patients with peritoneal ultrafiltration failure [10]. However, in idiopathic pulmonary fibrosis patients, although lymphatic density was found to be associated with disease severity, VEGF-C concentration in bronchoalveolar lavage fluid was lower than in healthy controls, possibly resulting from decreases in alveolar epithelial cells [74]. 

Targeting VEGF-C has been attempted to regulate lymphangiogenesis in animal experiments. The local adenoviral transfection of VEGF-C promoted lymphangiogenesis as well as VEGFR-3 expression in normal skin [98]. Moreover, it accelerated functional lymphatic regeneration after the surgical removal of axillary lymph nodes [91] as well as blood vessel enlargement and leakage in the skin and mucous membrane [99]. In addition, systemic treatment with an adenovirus vector expressing VEGF-C was found to attenuate intestinal inflammation and improve colitis in mice. Enhanced lymphangiogenesis induced by VEGF-C also accelerated the removal of inflammatory cells from inflamed colon tissue [97]. In an animal fibrotic disease model, VEGF-C was also used therapeutically via enhancing lymphangiogenesis. Hasegawa et al. described that intraperitoneal micro-osmotic pump delivery of recombinant human VEGF-C could promote total lymphatic density and reduce interstitial fibrosis and inflammation in the obstructed kidneys of mice [55]. In heart, Henri et al. described that intramyocardial delivery of VEGF-C_C152S_ by albumin–alginate microparticles improved cardiac edema and alleviated fibrosis and remodeling in post-myocardial infarction rats [72]. Notably, the therapy did not change the lymphatic density in the infarcted area, but high-dose VEGF-C increased the number of lymphatic capillaries in the subepicardium, while low-dose VEGF-C had the effect of altering the pre-collector lymphatic vessel size [72]. 

## 5. TGF-β Mediates Lymphangiogenesis during Fibrosis

As one of the most well-known pro-fibrotic factors, TGF-β is variously involved in organ fibrosis, sclerosis, and tumor bioactivity. In recent years, the effect of TGF-β on the structure and growth of lymphatic vasculature has been gradually explored.

TGF-β was found to exert an inhibitory effect on LECs, according to several studies. Exogenous TGF-β1 was observed to directly suppress the expression of Prox1 and LYVE-1, as well as the migration and tube formation in human LECs in vitro; all three isoforms of TGF-β were found to inhibit LEC proliferation [12,13,100]. Treatment with the TGF-β receptor I (TGF-βI) inhibitor LY364947 could mitigate the negative effect of TGF-β1 on LECs [12]. There are also reports that the presence of exogenous TGF-β1 reduced VEGF-C’s effects on LECs [9,12]. In addition, the expression of lymphatic markers in mesenchymal-derived and adipose-derived stem cells was impeded in response to TGF-β1 [100,101]. Thus, local overexpressed TGF-β1 was also regarded to have an inhibitory effect in lymphangiogenesis in some animal models. In a thioglycollate-induced peritonitis mouse model, higher diaphragmatic lymphatic density was induced, even though lower VEGF-C expression was seen in macrophages from the treated group [12]. In mouse lymphedema induced by tail excision, the use of collagen gel, which reduced fibrosis and TGF-β1 expression, could increase lymphatic regeneration in scarred areas as well as LEC proliferation in vitro. Further, supplementary TGF-β1 treatment impaired such effects and aggravated lymphedema [13]. Tail lymphedema could also be decreased by systemically or locally blocking TGF-β1 [14].

In LECs, TGF-β1 treatment had little effect on the production of VEGF-C and VEGF-D [13]. However, VEGF-C expression was found to be significantly upregulated in response to TGF-β in renal tubule cells, peritoneal mesothelial cells, macrophages, and fibroblasts [8,9,10,12,102] (Figure 1). Treatment with LY364947 reduced VEGF-C expression in these cell types. VEGF-C levels were found to be correlated with TGF-β levels in the effluent of PD patients and some tumor samples [10,15]. Furthermore, increased VEGF-C and lymphangiogenesis levels were observed in several fibrosis disease models. During UUO, the increased VEGF-C expression in proximal tubule cells and collecting duct cells was considered to be at least partially attributed to TGF-β1, which was expressed mainly in tubular epithelial cells [8]. The injection of LY364947 via the aorta suppressed VEGF-C expression in obstructed kidney as well as lymphangiogenesis [8]. Also, in a rat CG-induced peritoneal fibrosis model, intraperitoneal treatment with LY364947 also showed an anti-lymphangiogenic effect in the diaphragm [10]. Likewise, it was reported that TGF-β enhanced VEGF-C expression in some tumor cells [15]. VEGF-C was found to weaken the inhibitory effect of TGF-β1 in LECs and still promote lymphangiogenesis at higher concentrations [15]. It was also reported that treatment with VEGF-C reduced TGF-β expression in obstructed kidney [55]. Thus, it was considered that in vivo lymphangiogenesis developed, even though TGF-β had inhibitory effects on LECs as a consequence of upregulated VEGF-C [9,15].

TGF-β was also reported to promote hyaluronan expression in macrophages, synoviocytes, and fibroblasts [80,103,104], which bound to LECs and had a pro-lymphangiogenic effect, as described above. Hyaluronan was also found to induce VEGF-C production in macrophages [80]. Therefore, the accumulation of hyaluronan and increased TGF-β during fibrosis may lead to the progression of lymphangiogenesis. In human lung fibroblasts, it was reported that TGF-β1 upregulated VEGF-A and VEGF-C expression while downregulating VEGF-D, suggesting differences in TGF-β signaling in the regulation of these factors [102]. 

TGF-β is thought to induce phosphorylation of Smad2 or Smad3 in LECs as well as in tumor cells [12,15]. Elevated levels of phosphorylated Akt, p44/42 mitogen-activated protein kinase (MAPK), and heat shock protein (HSP) 27 were also observed in TGF-β1-treated fibroblasts [102]. The binding of Smad2 or Smad3 and VEGF-C promoter was demonstrated in tumor cells [15]. Therefore, it is expected that further elucidation of the TGF-β-regulated signaling pathway will provide new therapeutic targets for fibrosis-associated lymphangiogenesis.

## 6. Roles of CTGF in Fibrosis and Lymphangiogenesis

Connective tissue growth factor (CTGF, also known as CCN-2) is a member of the CCN (CTGF/Cyr61/Nov) family of matricellular proteins. Although a CCN-specific receptor has not yet been found, CCNs bind and modulate multi-ligands and receptors for other matricellular molecules [105]. CCNs play important roles in development, inflammation, cancer progression, tissue repair, and fibrosis [105]. CTGF is known as an important determinant of fibrotic tissue remodeling [106]. Further, CTGF is highly expressed in many fibrotic disorders, and plays a key role in ECM production and the profibrotic activities mediated by other growth factors [107,108]. CTGF is also known as a regulator of angiogenesis [109]. CTGF can promote angiogenesis in part by mediating TGF-β downstream and adhesive signaling such as integrins and heparin sulfate proteoglycan [110]. On the other hand, CTGF binds to VEGF-A and inhibits VEGF-A-induced angiogenesis [110,111,112]. The role of CTGF in angiogenesis may depend on tissue specificity and disease etiology.

CTGF is also involved in PD-associated peritoneal fibrosis. CTGF expression is increased in human PD effluents and human peritoneal biopsy samples in association with high peritoneal solute transport rate and UFF [113]. Peritonitis episodes markedly increase CTGF levels in human PD effluents [114]. CTGF is mainly enhanced in mesothelial cells and fibroblast-like cells in human fibrotic peritoneum with UFF [113]. CTGF production by human peritoneal mesothelial cells is regulated by GDP, AGE, and TGF-β [113,114,115]. Recent studies revealed that the inhibition of CTGF ameliorated CG-induced peritoneal fibrosis through the suppression of fibroblast accumulation, neoangiogenesis, and inflammation [116,117]. 

CTGF strongly contributes to the development and progression of chronic kidney disease (CKD) [118]. CTGF is overexpressed in various kidney diseases such as diabetic nephropathy [119], hypertensive nephrosclerosis [120], crescentic glomerulonephritis [121], and renal allograft fibrosis [122]. CTGF expression correlates with the severity of renal fibrosis [119,123]. TGF-β induces CTGF expression in multiple cell types, including mesangial cells and renal tubular epithelial cells [124,125]. On the other hand, CTGF modulates TGF-β signaling by direct physical interaction [107].

CTGF levels in plasma and urine were increased in type I diabetes patients with nephropathy [126,127]. Urinary CTGF correlated with urinary albumin excretion and glomerular filtration rate in patients with diabetic nephropathy [127]. Urinary CTGF also correlated with the progression of microalbuminuria, which is an early indicator of diabetic nephropathy [128]. In renal transplantation recipients, urinary CTGF levels were associated with the degree of interstitial fibrosis, and urinary CTGF expression at three months was associated with the progression of renal allograft fibrosis two years after transplantation [122]. We recently reported that tubulointerstitial CTGF expression (CTGFti) at three months was an independent predictor of interstitial fibrosis (IF) and tubular atrophy (TA) at five years after transplantation in stable renal transplant recipients [129]. Donor age is known to be a dominant predictor of histological damage. In predictive models, CTGFti data and urinary CTGF levels (CTGFu) at three months, in addition to donor age, improved the prediction of IF/TA at five years [129]. 

The reduction of CTGF is reported to be an effective antifibrotic strategy in experimental kidney diseases. Several reports showed that an approximately 50% reduction in CTGF was capable of reducing fibrosis development in moderate models of obstructive [130], diabetic [131], and allograft nephropathy [132], and in the remnant kidney model [133]. As for options for anti-CTGF therapy, FG-3019, a human monoclonal antibody against CTGF, has already been found to be safe and well tolerated in several clinical conditions [134,135]. A small phase-1 clinical trial was conducted in patients with diabetic nephropathy, and FG-3019 was found to significantly reduce albuminuria [134]. Unfortunately, a phase-2 study of FG-3019 in subjects with type 2 diabetic nephropathy was terminated early due to sub-optimal study design. However, phase-2 studies of FG-3019 in patients with idiopathic pulmonary fibrosis and other conditions are underway [135].

WNT1-inducible-signaling pathway protein-1 (WISP-1), another member of the CCN family, promotes VEGF-C production and lymphangiogenesis in human oral squamous cell carcinoma via the inhibition of microRNA-300 expression [136]. We recently clarified the involvement of CTGF in renal lymphangiogenesis [16]. We observed prominent lymphangiogenesis accompanying increased expression of CTGF and VEGF-C in human obstructive nephropathy. CTGF knockdown in mice resulted in decreased VEGF-C expression and lymphangiogenesis in parallel with a significant reduction of fibrosis in obstructive nephropathy and an ischemia reperfusion injury model [16]. In vitro, CTGF induced VEGF-C production in renal tubular cells; however, CTGF also directly bound to VEGF-C, thereby suppressing VEGF-C-induced LEC growth [16]. In vivo, not only intact CTGF, but also CTGF fragments can be detected. Unlike the full-length CTGF, these CTGF fragments did not affect VEGF-C function, which suggests that the direct inhibitory effect of CTGF on VEGF-C can be prevented, and may also be terminated by the cleavage of CTGF. Thus, CTGF appears to be directly involved in renal lymphangiogenesis through the regulation of VEGF-C production and activity.

## 7. Conclusions

Lymphangiogenesis is widely observed during the development of tissue fibrosis. One possible mechanism is that TGF-β promotes lymphangiogenesis through the TGF-β–VEGF-C pathway, which has been demonstrated in renal and peritoneal fibrosis. The role of fibrosis-associated lymphangiogenesis appears to depend on tissue specificity and disease etiology. However, clarification of specific downstream factors in the TGF-β–VEGF-C pathway is expected to lead to the development of new therapeutic strategies to combat the progression of tissue fibrosis and organ dysfunction in the future.

## Figures and Tables

**Table 1:** Lymphangiogenesis in fibrotic diseases.

Organs	Research Methods	Findings	References
Heart	Autopsied hearts after MI	・ Lymphangiogenesis was observed in healing stages with fibrosis.	[71]
	Rat models of MI	・ Administration of VEGF-C accereated lymphangiogenesis, leading to reducing cardiac inflammation, fibrosis, and dysfunction.	[72]
Lung	Human lung tissues and BALF	・ Areas of lymphatic vessels were correlated with the severeity of IPF. ・ Short-fragments of hyaluronic acid in BALF might mediate lymphatic endothelial cell growth. ・ CD11b^+^ alveolar macrophages in IPF could differenciate into lymphatic endothelial cells.	[74]
Kidney	Human kidney biopsies	・ The number of lymphatic vessels was correlated with the degree of tubulointerstitial fibrosis.	[49]
	Rat models of UUO	・ TGF-β1 promoted VEGF-C production in proximal tubular cells, collecting duct cells, and macrophages, leading to fibrosis-associated renal lymphangiogenesis.	[8]
	Cultured renal tubular cells, macrophages		
	Mouse models of UUO	・ TGF-β1 and TNF-α induced VEGF-C production in proximal tubular cells and macrophages. ・ VEGF-D prevented direct inhibitory effects on lymphatic endothelial cell growth by TGF-β1.	[9]
	Cultured renal tubular cells, macrophages, lymphatic endothelial cells		
	Rat models of proteinuric nephropathy	・ Suppression of lymphangiogenesis by anti-VEGFR3 antibody did not affect inflammation, fibrosis, and proteinuria.	[54]
	Mouse models of UUO, IRI	・ CTGF induced VEGF-C production in proximal tubular cells, leading to fibrosis-associated renal lymphangiogenesis. ・ CTGF bound to VEGF-C and inhibited VEGF-C-induced lymphatic endothelial cell growth.	[16]
	Cultured renal tubular cells, lymphatic endothelial cells		
	Mouse models of UUO	・ Administration of VEGF-C accereated lymphangiogenesis, leading to reducing inflammation, TGF-β1 expression, and fibrosis.	[55]
Peritoneum	Human peritoneal biopsies	・ Expression of lymphatic vessels was correlated with the degree of peritoneal fibrosis. ・ TGF-β1 promoted VEGF-C production in mesothelial cells, leading to fibrosis-associated peritoneal lymphangiogenesis.	[10]
	Rat PF models induced by CG		
	Cultured mesothelial cells		
	Mouse PF models induced by MGO	・ Suppression of lymphangiogenesis by soluble VEGFR-3 improved deteriorated net ultrafiltration.	[69]

MI: myocardial infarction; VEGF: vascular endothelial growth factor; BALF: bronchoalveolar lavage fluid; IPF: idiopathic pulmonary fibrosis; UUO: unilateral ureteral obstruction; TGF-β: transforming growth factor-β; TNF-α: tumor necrosis factor-α; VEGFR-3: VEGF receptor-3; IRI: ischemia reperfusion injury; PF: peritoneal fibrosis; CG: chlorhexidine gluconate; MGO: methylglyoxal.
